# Impact of Refinement on the Physicochemical, Structural, Functional, and Nutritional Properties of the Faba Bean (*Vicia faba* L.)

**DOI:** 10.3390/foods15213987

**Published:** 2026-11-06

**Authors:** Nneka Ajomiwe, Jaspreet Singh, Lovedeep Kaur

**Affiliations:** 1 School of Food Technology and Natural Sciences, Massey University, Palmerston North 4442, New Zealand; n.ajomiwe@massey.ac.nz (N.A.); j.x.singh@massey.ac.nz (J.S.); 2 Riddet Institute, Massey University, Palmerston North 4442, New Zealand

**Keywords:** faba bean derivatives, techno-functional properties, protein, DIAAS, rheology, thermal stability, FTIR

## Abstract

The faba bean (*Vicia faba* L.) is emerging as a sustainable protein source, with up to 32% protein and a high fibre content, comparable to that of mainstream plant proteins such as peas. This study assessed the physicochemical, thermal, techno-functional, and nutritional properties of faba bean flour (FBF), protein concentrates (FBPC), and protein isolates (FBPI). It aimed to determine whether protein functionality in faba bean derivatives is governed by matrix composition and processing methods or by protein purity alone. The unrefined and semi-refined fractions (FBF and FBPC) demonstrated superior solubility, stronger gelation, and enhanced emulsification and foaming stabilities, attributed to water-soluble albumins, native polysaccharides, and intact protein structures that promote intermolecular interactions. In contrast, FBPI showed greater water- and oil-holding capacities, but its reduced solubility and poor gel strength likely resulted from denaturation and the loss of functional components during processing. Thermal analysis further supported these findings, with higher denaturation temperatures in FBF indicating better stability. Despite increased essential amino acids with refining, valuable water-soluble albumins and non-essential amino acids were lost. While cultivar and processing differences may have contributed to the observed variations among commercially sourced faba bean derivatives, this study demonstrates that functionality extends beyond protein purity, highlighting the benefits of unrefined and semi-refined faba bean ingredients across various food applications.

## 1. Introduction

Plant proteins are increasingly recognised as sustainable protein alternatives owing to their lower environmental footprint and potential to improve food systems [1]. However, conventional protein isolation relies on wet fractionation processes, such as isoelectric precipitation, which require chemicals (such as NaOH, HCl, and hexane for oilseeds), increase resource use, reduce yield, and generate low-value side streams. Less-refined approaches, such as dry fractionation, offer improved sustainability by reducing water and solvent requirements while enabling whole-matrix utilisation [2,3].

In faba bean processing, studies have shown that increased refinement is linked to greater environmental impacts [4,5]. Whole faba bean flour has a lower carbon footprint (0.55–0.57 kg CO_2_ eq kg^−1^) than that derived from protein concentrate and isolate production, which require 13.7 and 54.9 MJ kg^−1^ protein, respectively, with isolates also consuming more water (13.4–47.7 kg kg^−1^ protein) [5]. Despite this, plant-based meat analogues often rely on protein isolates, which may lose functional components such as fibre and polysaccharides. The faba bean (*Vicia faba* L.), with its high protein content, adaptability, and nitrogen-fixing capacity, offers an opportunity for sustainable food applications [6]. Consequently, interest is growing in determining whether unrefined and semi-refined fractions can provide the functionality required for various food applications.

To the best of our knowledge, this study is the first to systematically compare the physicochemical, techno-functional, molecular, thermal, rheological and nutritional properties of commercial faba bean protein isolates (FBPI), protein concentrates (FBPC) and flour (FBF) within a single standardised framework. Previous studies have examined these ingredients in isolation or through fragmented comparisons [4,7]. To address this gap, the present study evaluates all three derivatives side by side and provides a comprehensive assessment of the relationship between commercial processing, ingredient structure and functionality. These fractions represent distinct functional matrices rather than simple differences in protein purity. Faba bean functionality largely depends on its major storage proteins, vicilin (7S) and legumin (11S), whose structural integrity can change during processing. The intensive wet extraction and thermal drying used in FBPI production have been reported to promote protein denaturation and functional losses, whereas milder dry milling and air classification preserve native macromolecular interactions [8]. Although FBPI typically contains 80–90% protein, its production yields low biomass (15–25%), requires large volumes of water, and incurs higher costs [9]. In contrast, FBF and FBPC retain native starch and fibre, which contributes to matrix functionality [10]. Removing native starch during protein isolate production and subsequently adding starch during food formulation is inefficient, increasing processing demands and material losses [11]. While highly purified isolates often show higher initial emulsification and foaming capacities due to rapid interfacial protein alignment, they frequently show lower long-term stability because they lack polysaccharide–fibre stabilising networks [12,13,14]. Conversely, whole flour and dry fractionated concentrates can exhibit superior emulsion stability and viscoelastic gelation at lower relative protein thresholds, driven by synergistic hydrocolloid effects from their intact polysaccharide and fibre matrices. These structural properties directly govern the final gelation, digestibility, and sensory attributes critical for consumer acceptance and targeted product development [15,16]. Thus, to address these processing and functional limitations, a comprehensive understanding of how commercial processing levels, alongside factors such as cultivar and growing environment, may influence ingredient functionality is essential for optimising food applications and industrial progress.

This comparative assessment serves as a strategic decision-making tool for ingredient selection based on scientifically validated structure–function relationships rather than on generic specifications. In addition, improving overall nutritional quality by reducing antinutritional factors through strategic processing remains paramount [17]. By using commercial samples, this study explored the structural integrity and techno-functional performances of these derivatives using advanced characterisation techniques [18], providing actionable data that directly addresses the scalability and application challenges often overlooked in bench-top extraction research, while supporting the industrial goal of recovering high-quality protein from sustainable side streams [19].

## 2. Materials and Methods

### 2.1. Materials

The raw materials utilised in this research were commercially acquired. Faba bean protein isolate, FBPI (87% protein content), Faba bean protein concentrate, FBPC (60.56% protein content), and Faba bean whole flour, FBF (32.05% protein content), on a dry basis, were purchased from NZ Proteins, Auckland, New Zealand; Beneo, Oreye, Belgium; and Woods Grain Pty Ltd., Queensland, Australia, respectively. Analytical-grade chemicals and reagents were used in all procedures. All analyses were carried out at the Nutritional Laboratory, Massey University (IANZ Accredited Testing Laboratory No 1363 926 927) and the Food Characterisation Laboratory, School of Food Technology and Natural Sciences (SFTNS), Massey University. Because this study uses commercial faba bean derivatives (FBF, FBPC and FBPI), the specific species and processing methods used to produce them are beyond the scope of this research.

### 2.2. Physicochemical Assessment

#### 2.2.1. Determination of Proximate Composition of Faba Bean Derivatives

The proximate composition of faba bean derivatives, protein isolates (FBPI), protein concentrates (FBPC), and flour (FBF), was determined by the Nutritional Laboratory, Massey University (IANZ Accredited Testing Laboratory No 1363 926 927), using standard AOAC methods. The crude protein contents of the samples were measured according to the AOAC 968.06 Dumas method (using a factor of 6.25), percentage moisture was determined by AOAC 925.10, 930.15, ash content by AOAC 920.153, total dietary fibre using Megazyme, AOAC 991.43, total starch by AOAC 996.11, and crude fat by AOAC 2003.06, with carbohydrates calculated by difference, according to AOAC methods [20].

#### 2.2.2. Colour Measurements

Colour is an important quality attribute in the food and bioprocess industries, influencing consumers’ choices and preferences [21]. Colour measurements of FB derivatives were performed using a handheld chroma meter (CR 400, Konica Minolta, Tokyo, Japan) in the CIE L*a*b* colour space. The instrument was calibrated prior to use. Colours were reported using the CIE parameters: L* (0 = black, and 100 = white), a* (greenness to redness), and b* (blueness or yellowness). The whiteness index was selected to enable comparability and to illustrate how the ingredients affect food systems [10]. Pairwise colour differences (ΔE*) were calculated between FBPI and FBPC, FBPC and FBF, and FBF and FBPI using the mean L*, a*, and b* values according to the equation below:

(1) $$\Delta E^{*}=\sqrt{{\left(L_{1}^{*}-L_{2}^{*}\right)}^{2}+{\left(a_{1}^{*}-a_{2}^{*}\right)}^{2}+{\left(b_{1}^{*}-b_{2}^{*}\right)}^{2}}$$

### 2.3. Tricine-Sodium Dodecyl Sulphate-Polyacrylamide Gel Electrophoresis (SDS-PAGE)

Tricine SDS-PAGE was conducted to characterise the proteins in raw faba bean derivatives (FPI, FBPC, and FBF) following the method of [22], with modifications. 100 mg of powder was dispersed in 1 mL of Milli-Q water. Thereafter, 5 μL aliquots were diluted with 95 μL of Milli-Q water. Each sample was mixed with an equal volume of Tricine sample buffer (Bio-Rad Laboratories, Auckland, New Zealand), with and without 2% β-mercaptoethanol, and heated at 60 °C for 10 min. Subsequently, 20 μL of sample and 10 μL of prestained protein standard (Precision Plus Protein™ All Blue, Bio-Rad, Auckland, New Zealand) were loaded into 4–20% Mini-PROTEAN^®^ TGX™ precast gels (Bio-Rad, Auckland, New Zealand). Electrophoresis was performed in 1× Tris/Tricine/SDS buffer (Bio-Rad, Auckland, New Zealand) at 125 V. Gels were then rinsed with RO water, stained with Bio-Safe Coomassie G-250 (Bio-Rad, Auckland, New Zealand) for 60 min, and destained in RO water for 30 min. Gel images were captured using a Molecular Imager Gel Doc XR system (Bio-Rad, Auckland, New Zealand) and analysed using Image Lab™ software (v6.0.0, Bio-Rad Laboratories, Hercules, CA, USA). Molecular weight distribution and band intensities were used to assess protein profiles under reducing and non-reducing conditions.

### 2.4. Fourier Transform Infrared (FTIR) Spectroscopy (Attenuated Total Reflectance)

The spectra of raw FB derivatives were obtained using a Fourier transform infrared (FTIR) spectrometer (Nicolet 5700, Thermo Fisher Scientific, Waltham, MA, USA), following the method described by [23]. Attenuated total reflection spectra were collected on a diamond crystal with 32 scans at a resolution of 4 cm^−1^. Omnic 9.2 (Thermo Fisher Scientific Inc., Waltham, MA, USA) and Peak Fit 4.12 software (SPSS Inc., Chicago, IL, USA) were used for baseline correction, smoothing, and Fourier self-deconvolution. Fourier self-deconvolution and second-order derivative fitting were used to quantitatively analyse the second-order derivative of the Fourier transform infrared spectra.

### 2.5. Techno-Functional Characterisation of Faba Bean (FB) Derivatives

#### 2.5.1. Water- and Oil-Holding Capacity (WHC and OHC)

The WHC and OHC of FB derivatives (FBPI, FBPC, and FBF) were determined using a method previously employed by [24], with slight modifications. A 9% (*w*/*w*) suspension was prepared for each sample. 0.5 g of the sample was dispersed in 5 g of reverse osmosis water/oil in a 50 mL screw-cap centrifuge tube. The dispersions were vortexed at room temperature for 10 s every 5 min for a total of 30 min, then centrifuged (Thermo Scientific Multifold X Pro series swing bucket rotor, Osterode am Harz, Germany) at room temperature at 2000× *g* for 30 min. The centrifuge speed was adjusted from the original to ensure adequate phase separation. The supernatant was carefully decanted, and the pellets were accurately weighed. The WHC or OHC was calculated as the ratio of the wet pellet weight to the sample’s initial weight, expressed as a percentage.

%WHC or OHC = (W/S) × 100(2)

where W is the weight of the wet pellet after decanting, and S is the weight of the sample used.

#### 2.5.2. Emulsifying Capacity and Stability of Faba Bean Derivatives

The emulsifying capacity (EC) and emulsion stability (ES) of faba bean flour, protein isolates, and concentrates were evaluated using the method of [25]. A 7% (*w*/*v*) suspension was prepared by dispersing 3.5 g of sample in 50 mL of water and homogenising (Heidolph DIAX 600, Schwabach, Germany) for 30 s at 24,000 rpm. Then, 25 mL of sunflower oil was added, and homogenisation was repeated for 30 s, followed by the addition of another 25 mL of oil and homogenisation for 90 s. The resulting emulsion was divided into two 50 mL centrifuge tubes and centrifuged (Thermo Scientific Multifold X Pro series swing bucket rotor, Osterode am Harz, Germany) at 1100× *g* for 5 min. EC was calculated as the percentage ratio of the emulsified layer volume relative to the total emulsion volume before centrifugation. For ES determination, the emulsions were heated at 85 °C for 15 min, cooled to room temperature, and centrifuged again at 1100× *g* for 5 min. ES was expressed as the percentage of EC retained after heating.

% Emulsion capacity (EC) = (A/B) × 100(3)

where A = volume of emulsified layer; B = volume of emulsion before centrifugation.

% Emulsion stability (ES) = (C/A) × 100(4)

where C = volume of emulsified layer after heating and centrifugation; A = volume of emulsified layer as recorded in %EC.

#### 2.5.3. Foaming Capacity and Foam Stability of Faba Bean Derivatives

The foaming capacity and stability of faba bean flour, protein concentrates, and isolates were measured according to the protocols described by [24], with slight modifications. Protein solutions (1%, *w*/*w*) were prepared in RO water based on the respective protein contents of the powders and stirred for 2 h at room temperature using a Heidolph MR Hei-standard stirrer to ensure homogeneity. Subsequently, 150 mL (V_i_) of each solution was transferred into a measuring cylinder and homogenised with a T10 basic S5 digital Ultra-turrax instrument (Thermo Fisher Scientific, Auckland, New Zealand) at 10,000 rpm for 3 min to generate foam. The resulting foam volume (V) was recorded, and the foam capacity (FC) was calculated using Equation (5). Foam stability (FS) was assessed by measuring residual foam volume after 30 min of storage at 25 °C and calculated using Equation (6). The modification from the original protocol was the use of RO water instead of 10 mM of sodium phosphate buffer (pH 7.00) and overnight stirring at 4 °C:

(5) $$\%\mathrm{Foaming}\mathrm{Capacity}\left(\mathrm{FC}\right)=\frac{V_{\mathrm{after}\mathrm{homogenisation}}-V_{i\mathrm{before}\mathrm{homogenisation}}}{V_{i\mathrm{before}\mathrm{homogenisation}}}\times 100$$

(6) $$\%\mathrm{Foaming}\mathrm{Stability}\left(\mathrm{FS}\right)=\frac{V_{\mathrm{foam}\mathrm{at}30\min}}{V_{\mathrm{initial}\mathrm{foam}}}\times 100$$

where V = volume, and V_i_ is the aliquot of protein solution used.

#### 2.5.4. Determination of Protein Solubility

Protein solubility was measured using the method described by [26], with slight modifications. A total of 2% (*w*/*w*) of the FB derivatives (flour, protein concentrates and isolates) were weighed into a 50 mL centrifuge tube and increased to 50 mL with RO (reverse osmosis) water. The resulting mixture was vortexed with a VELP ZX3 vortex mixer (VELP Scientifica, Velate, Italy) for 3 min, then continuously stirred under moderate conditions with a roller mixer (Digisystem Laboratory Instruments Inc., New Taipei, Taiwan) for 1 h. Next, the dispersions were centrifuged at room temperature at 17,000× *g* for 30 min. After centrifugation (Thermo Scientific Multifold X Pro series swing bucket rotor, Osterode am Harz, Germany), the supernatant was carefully removed and freeze-dried. The protein content of the supernatant and a 2% (*w*/*w*) solution of the various faba bean derivatives was measured according to the AOAC 928.08 Kjeldahl method (using a factor of 6.25). All the determinations were made in triplicate. Protein solubility was calculated using Equation (7).

(7) $$\%\mathrm{Protein}\mathrm{solubility}=\frac{\mathrm{Protein}\mathrm{content}\mathrm{of}\mathrm{supernatant}}{\mathrm{Total}\mathrm{protein}\mathrm{content}\mathrm{in}\mathrm{solution}}\times 100$$

#### 2.5.5. Least Gelation Concentration (LGC)

The minimum gelation concentration of FB derivatives (FBF, FBPC and FBPI) was determined using a method previously described by [25], with slight modifications.

Suspensions of each faba bean ingredient (flour, protein concentrate, and isolate) were prepared in Kimax glass tubes (DWK Life Sciences, Millville, NJ, USA) at concentrations ranging from 2% to 20% (*w*/*v*), in 2% increments, in 5 mL of RO water. The suspensions were vortexed for 15 s and then heated for 1 h in a water bath at 85 °C. This was followed by rapid cooling in ice for 5 min and further cooling at 4 °C for 2 h. The LGC is the % concentration above which the sample did not slip when the tubes were inverted.

### 2.6. Rheological Properties

The rheological properties of FBPI, FBPC, and FBF pastes were determined using the method of [27], with modifications. Samples were dissolved in water to obtain a paste containing 20% protein (*w*/*w*), achieving a concentration above the critical gelation point. The samples were then hydrated overnight at 4 °C, equilibrated to room temperature, and analysed on a Physica MCR 301 Modular Compact Rheometer (Anton Paar, Graz, Styria, Austria) equipped with a 40 mm parallel plate (1 mm gap). Strain sweeps (0.001–100% strain at 25 °C) were used to define the linear viscoelastic region (LVR), and a 0.01% strain level was selected for subsequent measurements. Temperature sweeps were conducted from 25 to 95 °C at 4 °C/min, held for 30 s, then cooled to 25 °C at the same rate. This was followed by frequency sweeps (0.01–10 Hz) at 0.01% strain within the LVR. All measurements were performed in triplicate and analysed using TA Universal Analysis software (v4.5A, TA Instruments, New Castle, DE, USA).

#### 2.6.1. Temperature Sweeps

Temperature sweeps were conducted to measure viscosity and assess the effects of heating and cooling on the materials. For this analysis, the pastes were loaded onto the rheometer plate, and mineral oil (Bio-Rad Laboratories, Rosedale, New Zealand) was applied along the plate edge to reduce moisture loss (drying) of the samples. The samples were then subjected to defined thermal and mechanical stresses. The temperature sweep analysis was carried out by increasing the temperature from 25 to 95 °C at 4 °C/min, with an angular frequency of 1 Hz and a strain amplitude of 0.001%, all of which were kept constant during the analysis. After holding at 95 °C for 30 s, samples were cooled from 95 °C to 25 °C at 4 °C/min and held for 30 s at 25 °C. The storage modulus (G′), loss modulus (G″), and tan δ were recorded at 36 points. All determinations were repeated three times.

#### 2.6.2. Frequency Sweeps

The same samples used for strain and temperature sweeps were used for the frequency sweep test. The viscoelastic properties were determined at 25 °C over a frequency range of 0.01–10 Hz, with 36 data points recorded.

### 2.7. Differential Scanning Calorimetry

The same sample preparation method used for the rheological test was also used for the thermal analysis of the FB derivatives (FBF, FBPC, and FBPI). The pastes containing 20% (*w*/*w*) protein were measured using differential scanning calorimetry (DSC Q2000 V24.11 Build 124, TA Instruments, New Castle, DE, USA), following the method of [28], with some modifications. The sample (14 mg) was sealed in hermetically sealed aluminium pans with lids (without pinholes) and analysed under an inert nitrogen atmosphere. Heating was performed from 20 °C to 120 °C at a rate of 5 °C min^−1^. A sealed, empty aluminium pan was used as the reference. Onset temperature (To), denaturation temperature (Td), and enthalpy of denaturation (∆H) were calculated from the thermograms using TA Universal Analysis software, Version V4.5A (TA Instruments, New Castle, DE, USA). The change from the original method was in the weight and heating range, as peaks were difficult to obtain at low sample sizes.

### 2.8. Amino Acid Analysis

The amino acid composition of samples of faba bean flour (FBF), protein concentrates (FBPC), and protein isolates (FBPI) was determined by the Nutritional Laboratory at Massey University (IANZ Accredited Testing Laboratory No 1363 926 927).

The amino acid profile (acid stable) was determined by HCl hydrolysis, followed by reversed-phase high-performance liquid chromatography (RP-HPLC) separation with AccQ Tag derivatisation (AOAC 994.12). CYS/MET was determined using performic acid oxidation (AOAC 985.28). Individual amino acid concentrations were quantified and expressed as mg per 100 mg of the sample. Eighteen amino acids were quantified, including essential and non-essential amino acids.

#### 2.8.1. Determination of Nutritional Parameters

The amino acid (AA) composition of the FBF, FBPC and FBPI samples was used to determine several nutritional parameters, as described by [6,29,30].

##### Amino Acid Score (Chemical Score)

The percentage of essential amino acids (EAA) in the sample divided by the % of essential amino acids (EEA) recommended by [31], reference an AA pattern (for children 6 months to 3 years).

##### Digestible AA, DIAA (Digestible Indispensable Amino Acid) Ratio and DIAAS (Digestible Indispensable Amino Acid Score)

Digestible amino acid (DAA) was calculated using Equation (8).

Amount of digestible AA (mg/g protein) DB = Individual AA (mg/g protein db) × True ileal digestibility (TID)(8)

AA digestibility coefficients determined by [29] for air-classified faba bean concentrate (63%) and faba bean starch concentrates (18.4% protein) were used to determine DIAAR.

DIAAS (%) and DIAA ratios for FBF, FBPC, and FBPI were calculated as described by [30] using Equations (9) and (10).

DIAAS (%) = Lowest value of the DIAA ratio × 100(9)

(10) $$\mathrm{DIAA}\mathrm{ratio}=\frac{\mathrm{mg}\mathrm{digestible}\mathrm{EAA}\mathrm{content}\mathrm{in}1g\mathrm{protein}\mathrm{of}\mathrm{the}\mathrm{sample}}{\mathrm{mg of the same dietary EAA in}\;1g\mathrm{of}\mathrm{the}\mathrm{reference}\mathrm{protein}}$$

### 2.9. Statistical Analysis

The reported data were analysed using IBM SPSS Statistics version 30 (IBM Corp., Armonk, NY, USA). Descriptive statistics were used to calculate means and standard deviations. Differences between samples were evaluated using one-way analysis of variance (ANOVA) for individual response variables and multivariate analysis of variance (MANOVA) for groups of related response variables. Where significant differences were detected, Tukey’s pairwise post hoc multiple comparison test was used to compare means and identify homogeneous subsets. Statistical significance was set at *p* < 0.05. All results are presented as mean ± standard deviation of three replicates. FTIR spectra were plotted using Origin 2023b (OriginLab Corporation, Northampton, MA, USA).

## 3. Results

### 3.1. Physicochemical Properties

#### 3.1.1. Proximate Composition of Faba Bean Derivatives

Table 1 presents the results of the proximate analysis of commercial faba bean derivatives, including protein isolates (FBPI), protein concentrates (FBPC) and flour (FBF).

The results clearly indicate a significant difference in crude protein content, with FBPI achieving the highest score (87.28%) and FBF the lowest (32%), consistent with expectations, given the refinement levels. Conversely, total carbohydrate and starch contents were the highest in FBF (55.65%, 40.13%) and lowest in FBPI (6.59%, 1.13%). Ash, fat, and total dietary fibre varied among the derivatives, with FBPC showing the highest values and FBF the lowest. It was anticipated that FBF would have the highest ash, fat, and total dietary fibre contents, but this was not the case. The semi-refined derivative (FBPC) exhibited the highest values.

#### 3.1.2. Colour

The colour results differed significantly across samples, as the flours’ brightness did not necessarily improve with refinement level, as shown in Table 2.

All the faba bean derivatives exhibited L* values (brightness) above 80%. Among the derivatives, FBPC had the highest L* value (approximately 97%), while FBPI had the lowest (88%). The a* values, representing the red–green axis, were negative for all samples, indicating a greenish hue, with FBPC the greenest and FBF the least green. For the b* values, reflecting the yellow–blue axis, FBPI showed the highest yellowness, whereas FBPC was the least yellow compared with FBF. The pairwise ∆E* values revealed the highest visual difference (8.38) between FBPI and FBPC. Conversely, FBF showed close colour matches with FBPI (4.20) and FBPC (4.30).

### 3.2. Molecular Characterisation by SDS-PAGE

**Figure 1:**
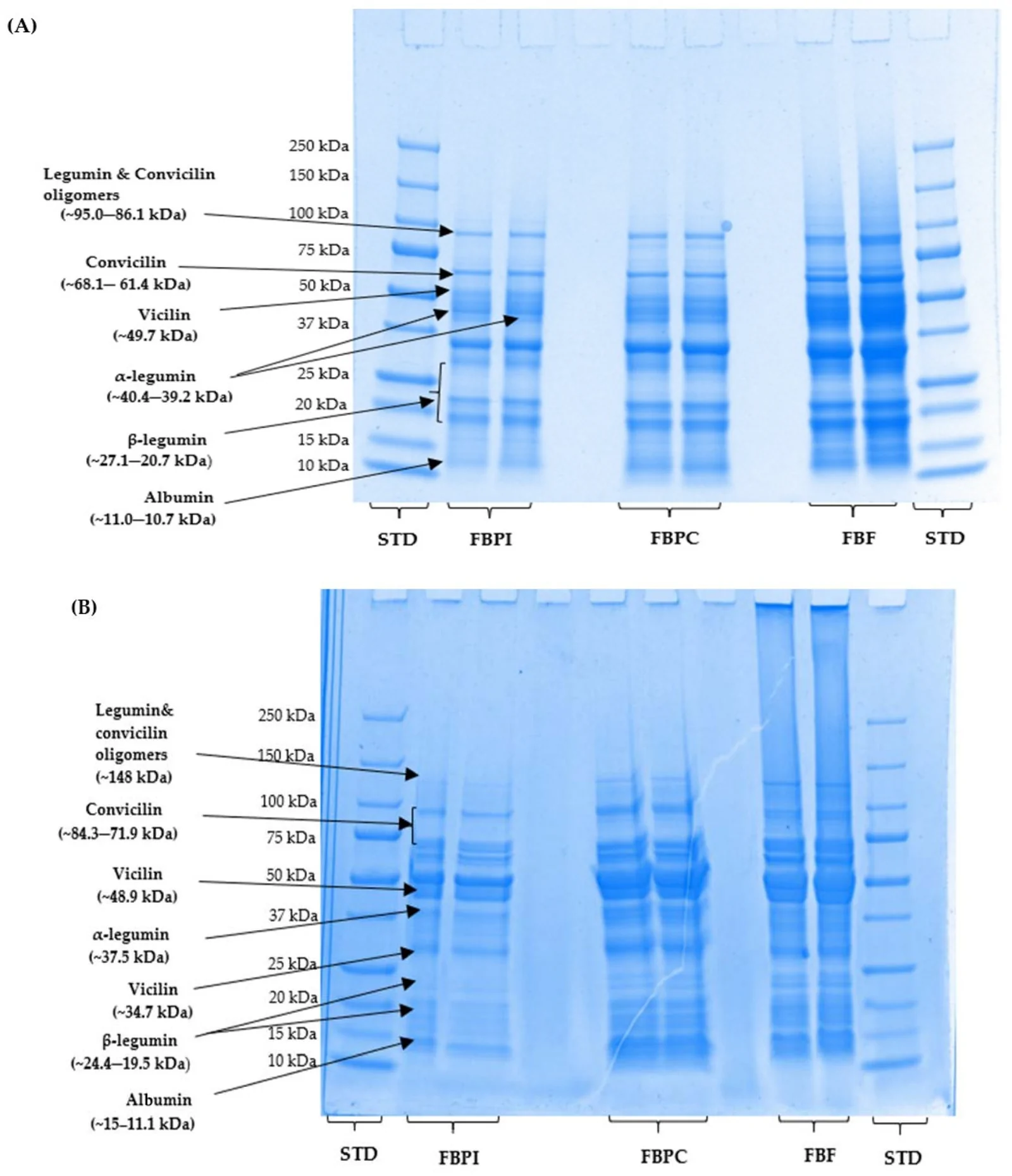
SDS-PAGE analysis of faba bean derivatives under (**A**) reduced conditions (with β-mercaptoethanol) and (**B**) non-reduced conditions (without β-mercaptoethanol).

SDS-PAGE was employed to analyse the protein profiles of faba bean flour (FBF), protein concentrate (FBPC), and protein isolate (FBPI) under reducing and non-reducing conditions. Figure 1 presents the protein profiles of FBF, FBPC, and FBPI.

The protein profile and molecular weight distribution were examined under both reduced and non-reduced conditions (Figure 1A,B). The gel results show that under reduced conditions, refinement from FBF to FBPI is evident. In this state, all FB derivatives contained globulin fractions such as vicilin (7S) and legumin (11S). FBF exhibited more smeared bands with β-mercaptoethanol, while FBPI had the least smearing. FBPC showed an intermediate profile. When treated with β-mercaptoethanol, legumin cleaved into α-legumin (~40.4–39.2 kDa) and β-legumin (~27.1–20.7 kDa) subunits. Convicilin (~68.1–61.4 kDa), vicilin (~49.7 kDa), and albumin (~11.0–10.7 kDa) were also detected, along with high-molecular-weight oligomers (~95.0–86.1 kDa). Compared with FBF and FBPC, FBPI lanes showed clearer, more intense bands for the main storage proteins, indicating removal of non-protein components, whereas in the non-reduced condition (Figure 1B), FBF and FBPC showed heavily smeared bands, suggesting the presence of non-protein constituents. Large legumin, convicilin oligomers (~148 kDa) and convicilin bands (~84.3–71.9 kDa) were also observed.

### 3.3. Fourier Transform Infrared (FTIR) Spectroscopy

**Figure 2:**
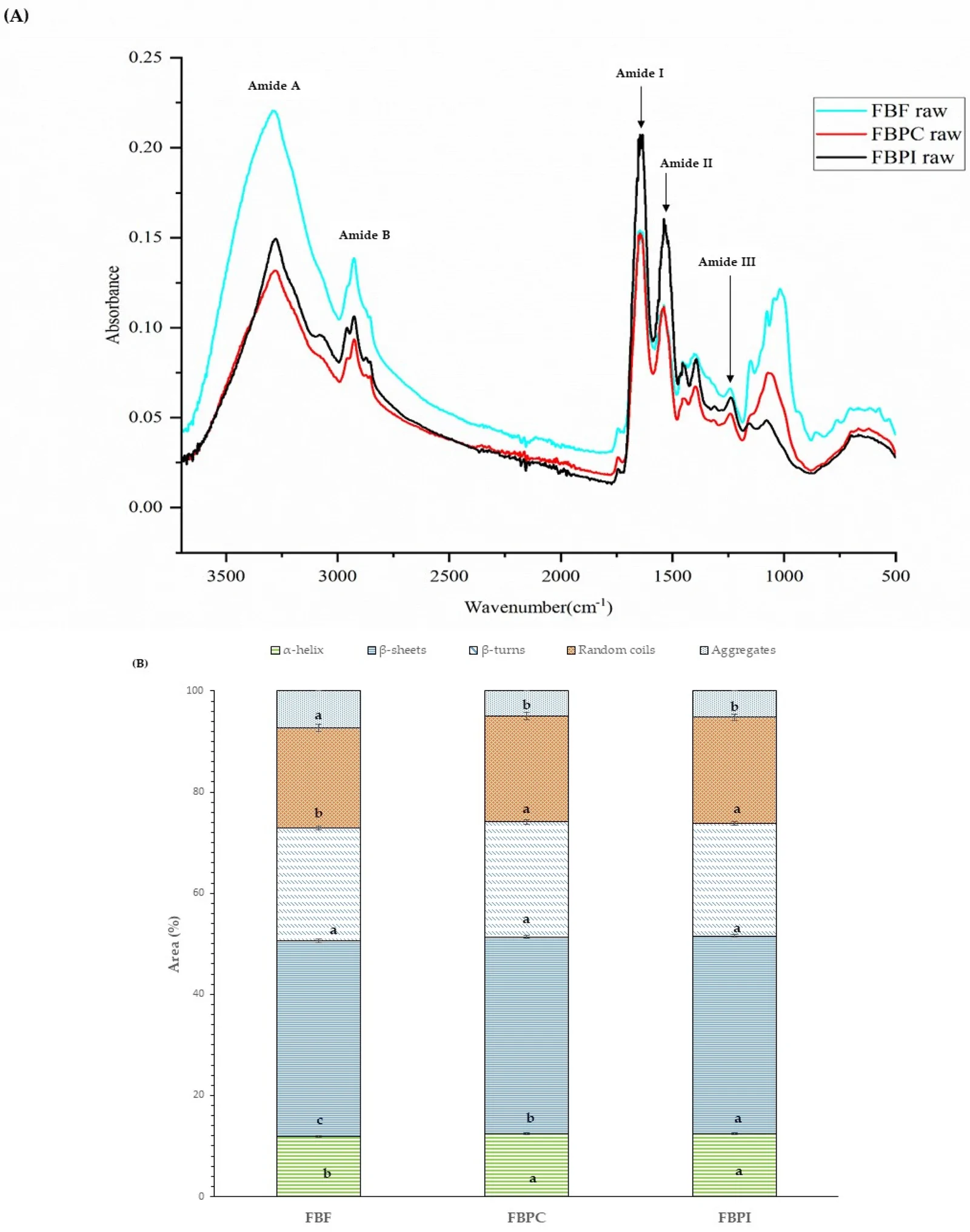
FTIR spectra (**A**) and secondary structure content (**B**) of raw faba bean derivatives (flour (FBF); protein concentrates (FBPC); protein isolate (FBPI)). Referenced locations of secondary structures: aggregated strands (1610–1620 cm^−1^), β-sheet (1620–1640 cm^−1^), random coil (1643–1650 cm^−1^), α-helix (1650–1660 cm^−1^) and β-turn (1660–1695 cm^−1^) [32]. All values are reported as the mean ± standard deviation of triplicate analyses. Values with different superscripts within the same column are significantly different (*p* < 0.05).

FTIR spectroscopy was used to evaluate molecular changes in faba bean fractions (flour, protein concentrates and isolates). The FTIR spectra of all samples are presented in Figure 2A,B.

The FTIR spectra display the key amide bands (I, II, III, A, and B), which are typical for protein samples. The amide I (approximately 1600–1700 cm^−1^) and amide II (around 1500–1600 cm^−1^) regions in the FTIR spectra reflect protein backbone vibrations and correlate with protein quantity and structure in the sample. FBPI showed the strongest FTIR peaks in the amide I and II regions, followed by FBPC and then FBF, suggesting a correlation between protein purity and FTIR signal intensity.

#### Secondary Structural Content of FB Derivatives

Table 3 presents estimates of secondary structures, including aggregated strands, α-helices, β-sheets, β-turns, and random coils, derived from the amide I region (1600–1700 cm^−1^). β-sheets were the most prevalent protein secondary structure across all samples, while aggregates were the least common. β-sheet content differed significantly among the samples (FBPI, FBPC, and FBF), with FBPI showing the highest proportion (39.11%) and FBF the lowest (38.67%). However, no statistically significant difference in β-turn content was found among the samples overall. For α-helices, random coils, and aggregates, no significant differences were observed between FBPI and FBPC; however, significant differences emerged when comparing FBPI and FBPC with FBF.

### 3.4. Techno-Functional Properties of Faba Bean Derivatives: Faba Bean Protein Isolates (FBPI), Faba Bean Protein Concentrates (FBPC) and Faba Bean Flour (FBF)

Table 4 presents the mean ± SD values of the techno-functional properties of faba bean derivatives (water- and oil-holding capacity, emulsifying and foaming capacity, emulsifying and foaming stability, and protein solubility).

#### 3.4.1. Water- and Oil-Holding Capacities

From the table above (Table 4), the water-holding capacities of the various faba bean derivatives varied significantly; faba bean protein isolates (FBPI) exhibited the highest water-holding capacity (WHC) compared with faba bean protein concentrates (FBPC) and faba bean flour (FBF). By contrast, oil-holding capacity followed conventional assumptions but differed from the trend observed for WHC. FBPI exhibited the highest oil-holding capacity, while FBF had the lowest.

#### 3.4.2. Emulsifying Capacity and Stability

The emulsifying capacities and stabilities varied significantly across all faba bean derivatives. However, the pattern reversed when comparing emulsifying capacity with stability. The emulsifying capacity was highest for faba bean protein isolates and lowest for faba bean flour. In contrast, faba bean flour showed the highest emulsification stability across all samples, with faba bean protein concentrates exhibiting intermediate performance in regards to both emulsification capacity and stability. Overall, as refinement increased, emulsification capacity improved, but emulsification stability declined.

#### 3.4.3. Foaming Capacity and Stability

There was a statistically significant difference in foaming capacity; faba bean protein concentrates (FBPC) had the highest foaming capacity, whereas the lowest value was recorded for faba bean flour (FBF), representing a 39% decrease relative to the mean value of the overall samples (FBPI, FBPC, and FBF). In contrast, foaming stability did not follow the same trend as foaming capacity; it decreased with increasing refinement (FBF > FBPC > FBPI).

#### 3.4.4. Protein Solubility

The commercial faba bean derivatives, protein isolate (FBPI), protein concentrate (FBPC) and flour (FBF), showed a decrease in protein solubility with increasing refinement (FBF > FBPC > FBPI). All three samples differed significantly from each other.

#### 3.4.5. Least Gelation Concentration

FBPI and FBPC performed similarly, with no statistically significant difference (12%, *p* < 0.05) in the least gelation concentration (LGC). A notable difference was observed among the derivatives, with FBF at 8% compared to FPBC and FBPI at 12%.

### 3.5. Rheological Properties of Faba Bean Derivative Pastes

The rheological properties of pastes made from faba bean derivatives, protein isolates (FBPI), protein concentrates (FBPC), and flour (FBF), with a 20% *w*/*w* protein content, were assessed using temperature and frequency sweeps at a strain of 0.01% within the linear viscoelastic region (see Supplementary Data, Figure S1).

#### 3.5.1. Temperature-Dependent Rheological Behaviour

**Figure 3:**
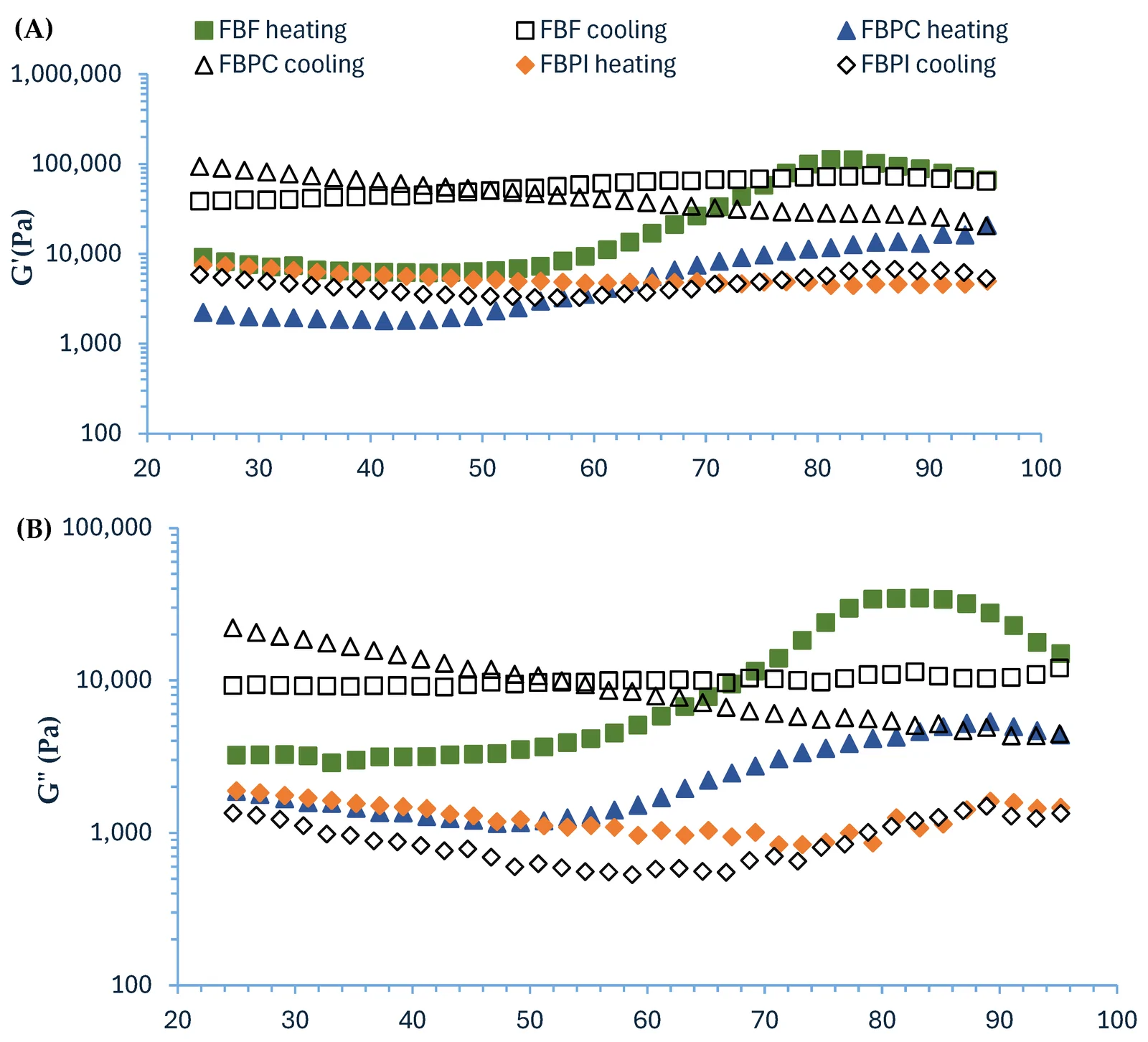

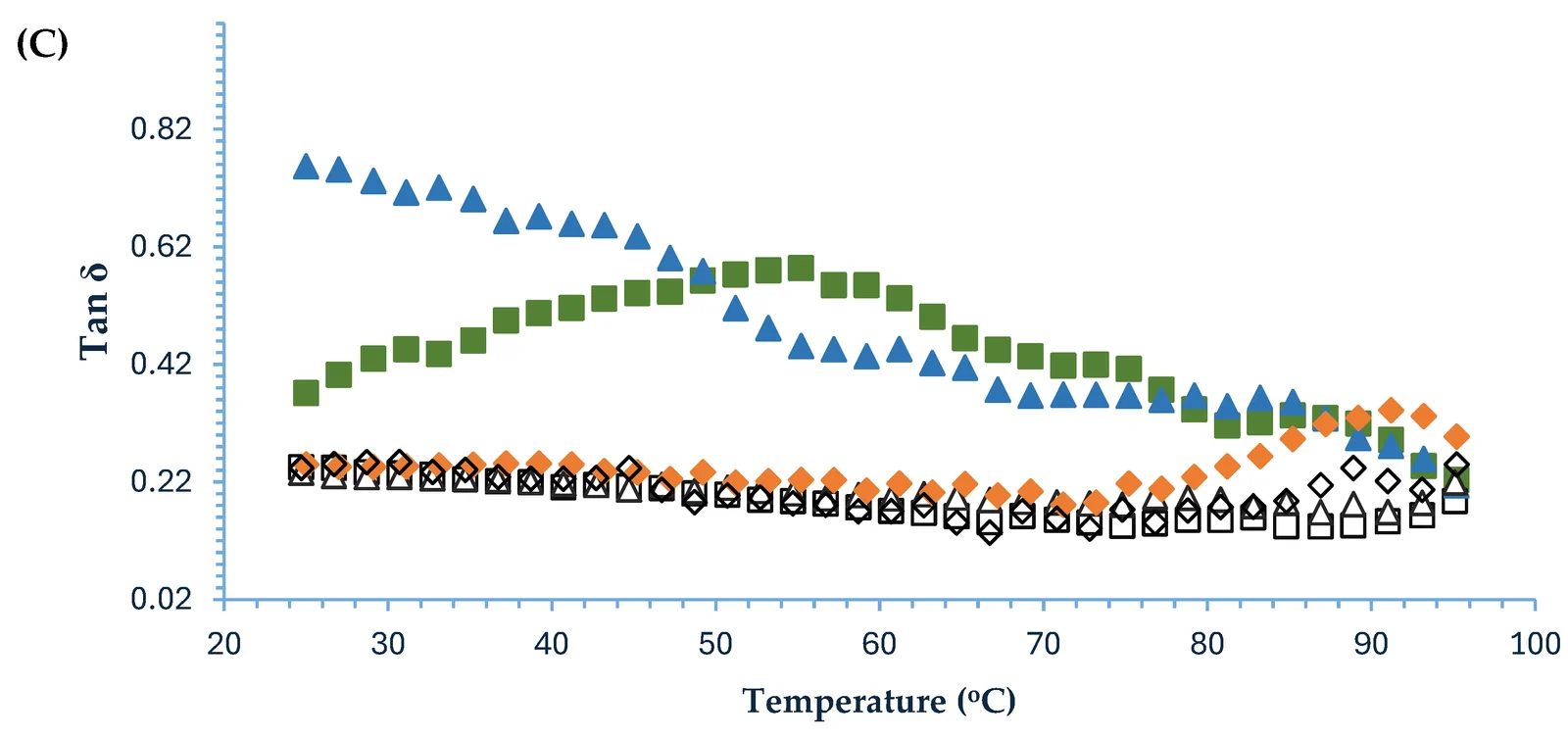
Viscoelastic properties of plant protein pastes during temperature sweeps. Faba bean flour (FBF); faba bean protein concentrate (FBPC); faba bean protein isolate (FBPI). (**A**) Storage modulus (G′); (**B**) loss modulus (G″); (**C**) tan δ. The results shown are the mean values of triplicate tests.

Temperature sweep tests on FBF, FBPC, and FBPI were conducted to assess their dynamic rheological properties. As shown in Figure 3A,B, all samples had elastic modulus (G′) values exceeding their loss modulus (G″), indicating mainly elastic behaviour across the temperature range (heating: 25–95 °C; cooling: 95–25 °C). FBF began with a G′ value like that of the isolates, with an onset temperature of around 52 °C, then rose sharply to 112,700 Pa at approximately 81 °C. FBPC started lower, with a G′ of about 2230 Pa. During cooling, the storage and loss moduli of all paste samples increased steadily as temperature decreased, though FBF showed a slight decrease. The G′ values (heating and cooling) of FBPI remained relatively constant, with a crossover at 66.7 °C, while its G″ value was considerably lower than that of FBPC and FBF. In Figure 3C, during heating, FBPC showed the highest tan δ, followed by FBF, with FBPI having the lowest. As the temperature rose, tan δ decreased for FBPC, whereas it initially increased and then decreased for FBF and FBPI.

#### 3.5.2. Frequency-Dependent Rheological Behaviour

**Figure 4:**
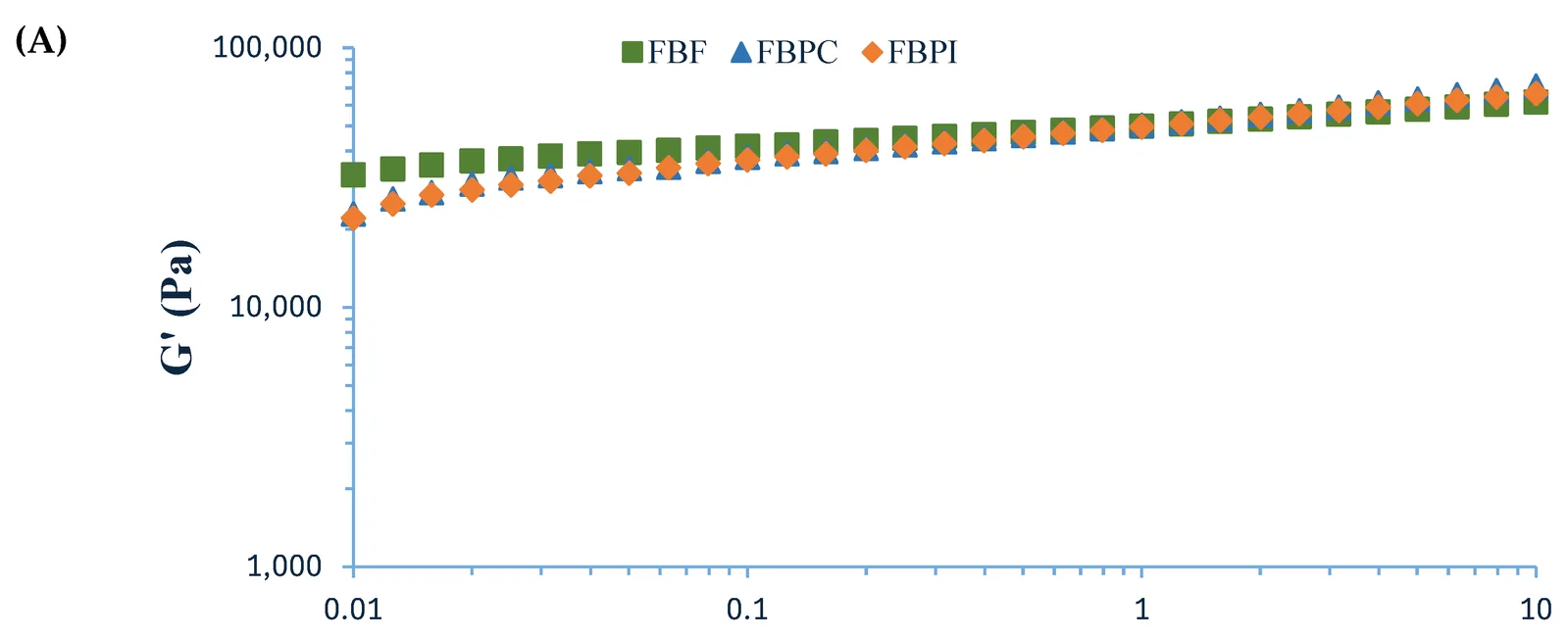

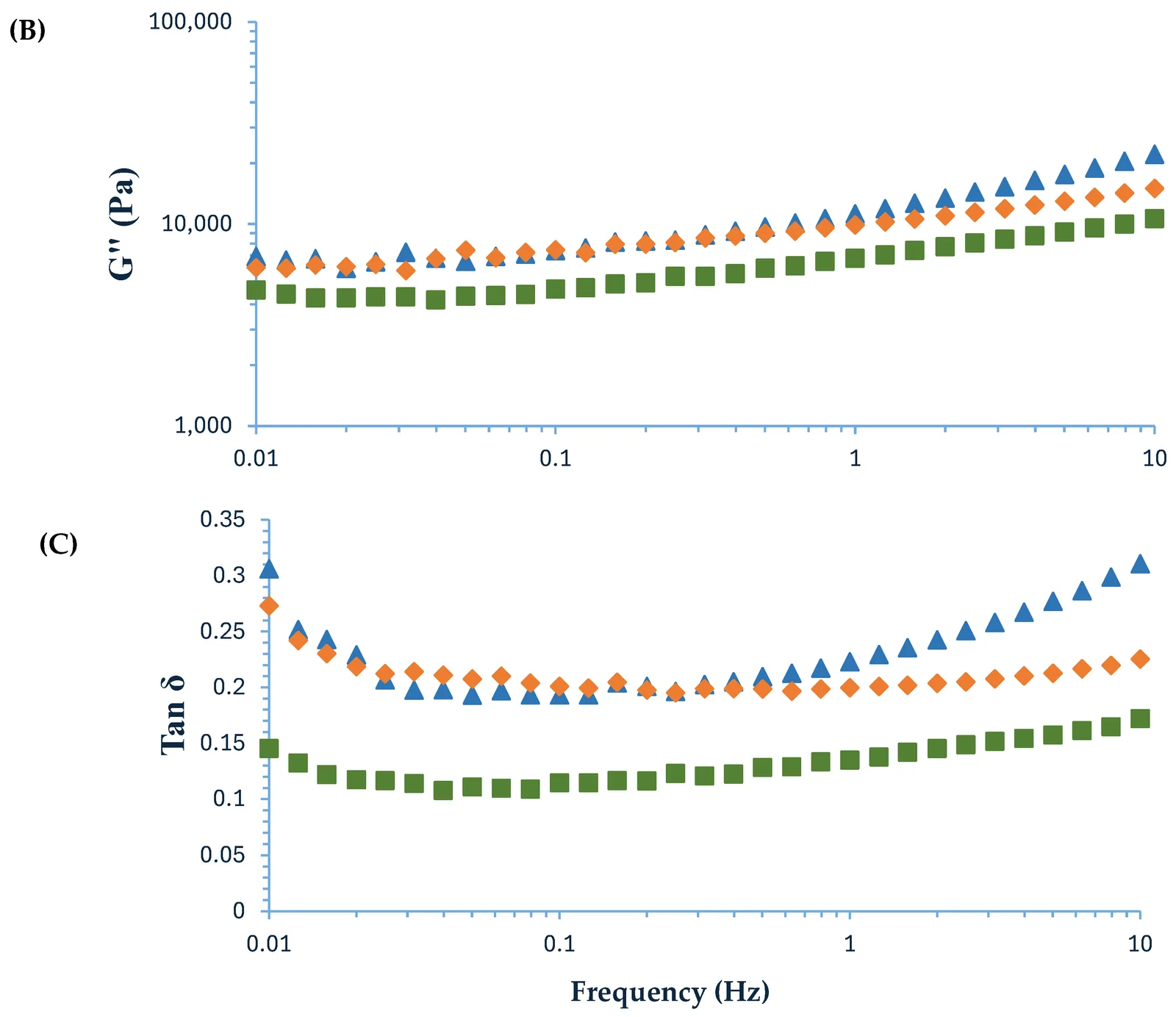
Viscoelastic properties of plant protein pastes during frequency sweeps (from 0.01 to 10 Hz). Faba bean flour (FBF); faba bean protein concentrate (FBPC); faba bean protein isolate (FBPI). (**A**) Storage modulus (G′); (**B**) loss modulus (G″); (**C**) tan δ. The results shown are mean values from triplicate tests.

The frequency sweep analysis of FB derivative pastes is illustrated in Figure 4.

Oscillatory frequency sweep tests were performed to assess the viscoelastic properties of faba bean flour (FBF), protein concentrate (FBPC), and protein isolate (FBPI). All samples showed gel-like behaviour, with the storage modulus (G′) higher than the loss modulus (G″) across the frequency range of 0.01–10 Hz, indicating mainly elastic characteristics. At lower frequencies (0.01–0.40 Hz), FBF had the highest G′ values (46,300 Pa) compared to those of FBPC and FBPI, suggesting a more rigid, elastic structure in this range. Above 0.40 Hz, FBF’s G′ value decreased more than that of either FBPC or FBPI, with FBPC reaching a peak G′ of 71,200 Pa at 10 Hz. The behaviour differed for the loss modulus and damping factor: FBF maintained the lowest G″ value throughout the testing range, while initially, FBPC and FBPI had similar G″ values, but as frequency increased, FBPC surpassed FBPI, ending with the highest G″ value and tan δ of 10,220 Pa and 0.311, respectively.

### 3.6. Thermal Properties

The thermal stability profiles of faba bean flour (FBF), faba bean protein concentrate (FBPC), and faba bean protein isolate (FBPI), determined by differential scanning calorimetry (DSC) at a heating rate of 5 °C/min and presented in Table 5, provide important insights into how composition and processing affect protein denaturation behaviour.

FBPC and FBPI showed single denaturation peaks at approximately 98 °C and 90 °C, respectively. FBPC had an elevated denaturation temperature (T_d_) and a relatively high enthalpy change (1.30 ± 0.33) J/g, with an onset temperature (T_o_) of 83.75 ± 1.30 °C and an end temperature (T_e_) of 118.10 ± 0.04 °C. Although FBPI had a similar Te to that of FBPC, it had the lowest enthalpy change (ΔH) and showed no statistically significant differences in onset and end transition points compared with those of FBPC. FBF displayed two distinct endothermic transitions at 73 °C and 100.34 °C, with onset and end temperatures significantly different from those of FBPC and FBPI. Regarding ΔH, the first peak differed significantly from those of FBPI and FBPC, but the second peak’s enthalpy change did not differ significantly from that of FBPI.

### 3.7. Amino Acid (AA) Profiling

Faba bean (*Vicia faba* L.) derivatives, flour (FBF), protein concentrates (FBPC), and protein isolates (FBPI), were analysed for their amino acid profiles. Table 6 presents the standard 18-amino-acid mixture for these derivatives. This study found glutamic acid to be the most abundant amino acid, with average concentrations ranging from 5.10 to 15.15 mg per 100 mg of sample across FBF to FBPI. Taurine was the least abundant amino acid, with FBF at 0.04 mg/100 mg of sample, FBPC at 0.02 mg/100 mg of sample, and FBPI at 0.03 mg/100 mg of sample. As expected, the contents of essential and non-essential amino acids increased with refinement. The limiting sulphur-containing amino acid score (AAS*) of faba bean derivatives increased with refinement. FBPI had an AAS of 81.47%; FBPC had 76.16%; and FBF had the lowest value (66.14%). The digestible indispensable amino acid scores (DIAAS) of faba bean derivatives were estimated using the published reference AA pattern for the 0.5- to 3-year-old age group [30] and the digestibility coefficient reported by [28]. In this study, FBF had a DIAA ratio of 0.54 and a DIAAS (adjusted % digestibility) of 56.44%; FBPC had a DIAA ratio of 0.62 at 100% digestibility and a DIAAS (adjusted % digestibility) of 62.11%. However, the DIAA ratio and DIAA score for FBPI could not be calculated because no reference ileal digestibility data were available for the individual essential amino acids.

## 4. Discussion

### 4.1. Proximate Composition of Faba Bean (FB) Derivatives (Flour, Protein Concentrates and Isolates)

The proximate composition of FB derivatives is shown in Table 1. Moisture content increased across faba bean protein isolates (FBPI), protein concentrates (FBPC), and flour (FBF), indicating a progressive reduction in moisture as protein purification increased. This result is comparable to previously reported values for faba bean flour, protein concentrates, and isolates [5,33,34]. Similar results for FBF protein content, i.e., 29.09%, 57.6%, and 87.0% for FBPC and FBPI, respectively, have been reported previously [8]. Starch content also decreased significantly from 40.13% in FBF to 5.80% in FBPC and 1.13% in FBPI (*p* < 0.05). Given the role of starch gelatinisation and gel structure, these differences may have influenced the gelation properties of the ingredients, particularly the superior gelation behaviour observed for FBF. In this study, FBPC had almost double the fat content of FBF, while FBPI had the lowest fat content. Previous studies reported similar results [8,33,35]. FBPC had significantly higher total ash content than that of either FBF or FBPI. Reference [36] reported a similar FBPC ash content of 5.2 g/100 g. Other authors reported that the FBPI ash content (9.0% *w*/*w*) was higher than that in FBPC (5.4% *w*/*w*), which may be due to the addition of alkali or acids during protein isolation, leading to salt formation and increased ash content. However, the higher ash content in pulse flour suggests it could boost mineral intake when incorporated into food products alongside low-ash wheat flour. The ash content of the FBPI used in this study was approximately 3%, which may be due to the seed variety or the processing method [36].

### 4.2. Colour Comparison

As shown in Table 2, all faba bean derivatives exhibited L* values (brightness) above 80%. Among the derivatives, FBPC had the highest L* (approximately 97%), while FBPI had the lowest, resulting in a darker appearance. The a* values, representing the red–green axis, were negative for all samples, indicating a greenish hue, with FBPC the greenest and FBF the least green. For the b* values, reflecting the yellow–blue axis, FBPI showed the highest yellowness, whereas FBPC was the least yellow, followed by FBF. Differences in colour parameters among the derivatives may reflect the extent of pigment retention and the removal of non-protein components during processing, altering the levels of phenolic compounds, pigments, and other non-protein substances that contribute to the visual qualities of legume-based ingredients. The pairwise total colour differences were calculated, and the largest difference occurred between FBPI and FBPC. The identical colour observed when FBF was compared with FBPI and FBPC demonstrates that extraction may introduce greater optical divergence than that caused by the initial fractionation from whole flour [10,37,38,39]. From a functional perspective, the lighter appearance of FBPC may benefit its incorporation into light-coloured food products. In contrast, the higher yellowness of FBPI could limit its suitability for applications where colour uniformity is essential. Overall, the colour analysis emphasises how processing affects the visual quality of faba bean-based ingredients, which is essential for their application in food products.

### 4.3. SDS-PAGE Analysis of Faba Bean Derivatives

Faba bean proteins include legumin, vicilin, and convicilin, with globulins comprising 70–80% of the protein content, albumins 10–20%, and prolamins and glutelins less than 5% [40]. Legumin constitutes about half of the protein, with vicilin and convicilin together accounting for about 27%, with variations among genotypes [39]. Legumin is a hexamer of six α-β monomers (~60 kDa each) linked by disulphide bonds and rich in cysteine and methionine. Vicilin proteins are trimeric (~50–60 kDa), stabilised by hydrophobic interactions, and low in cysteine. Convicilin (~220–290 kDa), a 7S pulse globulin, is less common, sharing a core structure with vicilin but featuring a highly charged, hydrophilic N-terminal extension and a different amino acid profile that includes sulphur residues but no carbohydrate side chains [41]. Under non-reducing conditions, bands at ~15 kDa and ~11 kDa were attributed to albumins, while bands at ~24.4 kDa and ~19.5 kDa corresponded to β-legumin. Bands at ~37.5 kDa corresponded to α-legumin, ~48.9 kDa to vicilin, and ~71.9 to ~84.3 kDa to convicilin. Higher-molecular-weight bands above 100 kDa likely represented protein aggregates stabilised by disulphide bonds. Under reducing conditions, these bonds were broken, revealing smaller subunits, consistent with findings of disulphide-linked complexes in faba bean isolates [39,42]. Distinct bands were observed under reducing conditions, highlighting 11S legumin, with α-legumin (~40.4–39.2 kDa), β-legumin (~27.1–20.7 kDa), and convicilin (~68.1–61.4 kDa). Low-mass components (~10.7–11.0 kDa) represented functionally significant fractions. Bands up to ~148 kDa indicated oligomers or aggregates, with reduced high-molecular-mass species in FBPI and FBPC suggesting improved globulin purity following extraction [39,43]. These observations were consistent with the DSC results, which showed higher denaturation temperatures for FBPI and FBPC, indicating more-stable proteins than those in FBF. Overall, these findings underscore the heterogeneity of faba bean proteins and show that isolation procedures for concentrates and isolates enrich globulin fractions (vicilin and legumin) while reducing non-globulin components to improve purity and function [10,39,43,44].

### 4.4. Fourier Transform Infrared (FTIR) Spectra

The FTIR spectra trend reflects the effectiveness of the fractionation process in enriching protein content: as protein purity increases from flour to concentrate to isolate, the corresponding FTIR amide I and II signals become more pronounced. The higher peak intensities in faba bean protein isolates (FBPI) indicate a greater abundance of protein secondary structures detected in these spectral regions, and this pattern aligns with the expected composition of each fraction [8,45,46]. The position of the amide I band depends on the protein’s backbone conformation and hydrogen-bonding features, mainly indicated by the stretching vibrations of the C=O and C=N groups. Amide I is affected by the number and strength of hydrogen bonds, lowering its natural frequency. Hydrogen bonding also influences the amide II vibration, making it highly sensitive to these bonds [32].

#### Secondary Structures in Amide I Region

The higher proportions of α-helix and random coil structures in FBPI and FBPC compared with FBF likely reflect protein enrichment and purification during fractionation, which reduce non-protein components such as starch and fibre that can promote protein aggregation and structural disorder [45,46]. Consistent with previous reports, fractionation methods, especially mild or hybrid approaches, have been shown to preserve native protein secondary structures, resulting in similar α-helix and random coil content in both FBPI and FBPC, whereas FBF, with its more complex matrix and lower protein purity, tends to exhibit more aggregated and less ordered protein structures [47,48]. This observation correlates with the results in Section 3.4.4 and Table 4, which show FBF having the highest protein solubility compared with that of FBPI. The subtle differences in β-sheet content and the lack of significant changes in β-turns suggest that these structural elements are less affected by fractionation, possibly because β-sheets are the dominant and more stable structures in faba bean proteins [46,49]. The higher levels of aggregate structures in FBF further support the influence of protein–starch–fibre interactions on protein organisation. Overall, variations in secondary structure among faba bean ingredients may explain their different solubilities.

### 4.5. Techno-Functional Properties of Faba Bean (fb) Derivatives: Faba Bean Protein Isolates (FBPI), Faba Bean Protein Concentrates (FBPC) and Faba Bean Flour (FBF)

Table 4 presents the mean ± SD values for the techno-functional properties of faba bean derivatives (water- and oil-holding capacity, emulsifying and foaming capacity, emulsifying and foaming stability, and protein solubility).

#### 4.5.1. Water-Holding Capacity of Faba Bean Derivatives

The water-holding capacity (WHC) reflects the ability of protein flour to absorb water without dissolving [50]. FBPI exhibited a higher WHC than did either FBPC or FBF, likely due to its greater surface charge and hydrophilicity, which facilitate hydration of protein matrices, as well as the processing conditions used during protein extraction [51,52]. Although FBF has less protein, it showed a higher WHC than that of FBPC, probably because of its high starch, carbohydrate and fibre contents. Fibre facilitates water absorption through hydrophilic interactions, while hydrogen bonding between starch hydroxyl groups (amylose and amylopectin) and water molecules further contributes to FBF’s higher WHC. The corresponding secondary-structure results revealed that FBPI had the highest β-sheet content, whereas FBF had the lowest β-sheet and α-helix contents. This increase in β-sheet content correlates with higher WHC and improved gel properties, as more ordered β-sheet structures facilitate the formation of compact, homogeneous networks that restrict water mobility and increase bound water [53,54,55]. Differences in protein secondary structure may also alter the exposure of hydrophilic and hydrophobic groups on protein surfaces, which may influence the WHC of the ingredients [48]. Although FBPC has higher α-helix and β-sheet contents than those of FBF, one would expect its water-holding capacity (WHC) to be higher; however, it was lower, suggesting that non-protein components in FBF contributed more to water absorption and retention than did the protein secondary structures [11,56].

#### 4.5.2. Oil-Holding Capacities (OHC) of FB Derivatives

Oil-holding capacity (OHC) is the ability of flour to bind fat through capillary attraction, hydrophobic interactions, surface area, and the arrangement of hydrophobic amino acids, which is dictated solely by secondary and tertiary structures [35]. OHC acts as a flavour-retainer and enhances the mouthfeel of foods. It is regarded as a key functional property in food applications [50,57]. In this study, FBPI exhibited a higher OHC than did either FBPC or FBF, consistent with previous findings [35] stating that, in faba bean proteins (FBPs), the isolates showed the highest OHC, followed by similar values for the concentrate and de-flavoured samples. This increase in the OHC of FBPI, as shown in Table 4, may be attributed to partial protein denaturation during extraction, which exposes hydrophobic regions, and to the higher concentration of FBPI, which enhances the stability of the oil–water interface [40,58]. Additionally, protein–lipid interactions involve hydrophobic, electrostatic, and hydrogen-bond interactions. Hydrophobic interactions play a crucial role in the stability of protein-lipid complexes, so proteins with more hydrophobic regions exhibit better OHC [59]. Changes in β-sheet and unordered structures can expose more hydrophobic sites, enhancing oil binding. Since non-protein components like starch and fibre do not bind oil as effectively as proteins, the role of protein structure is more pronounced for OHC than for WHC [35,60].

#### 4.5.3. Emulsifying Capacity (EC) and Stability (ES) of FB Derivatives

The emulsifying capacity and stability of plant-based emulsifiers depend on form (isolate, concentrate, or flour), processing methods, and modifications, each of which influences their functional performance in emulsions. As shown in Table 4, the emulsifying properties of the faba bean derivatives varied considerably. Faba bean protein isolate (FBPI) exhibited the highest emulsifying capacity (EC) but the lowest emulsion stability (ES), indicating a strong ability to form emulsions that are not sustained over time. In contrast, faba bean flour (FBF) produced the most stable emulsions, despite having a lower EC. Faba bean protein concentrate (FBPC) exhibited moderate EC and ES values, indicating balanced emulsification performance. The observed differences in emulsifying properties among the faba bean derivatives may be attributed to their compositional and functional characteristics [53]. The high EC of FBPI is likely due to its elevated protein content, which increases the availability of surface-active molecules for rapid adsorption at the oil–water interface. However, lower solubility and a greater tendency to aggregate after isoelectric precipitation may hinder the formation of a cohesive interfacial film, thereby reducing ES [61]. Conversely, the higher levels of carbohydrates and other non-protein components increase the viscosity of the continuous phase and act as physical barriers to droplet coalescence, thereby improving ES [5,35,36]. Differences in thermal behaviour further support these observations. FBPI, with a denaturation temperature around 90 °C, is highly purified and features more accessible, flexible protein structures. This enables rapid adsorption at oil–water interfaces and yields the highest emulsification capacity. In contrast, concentrates, with a higher denaturation temperature of about 98 °C, tend to have more stable or aggregated protein structures that can slightly reduce interfacial activity relative to that of isolates, resulting in a moderate emulsification capacity [35,46]. Flour exhibited two DSC peaks at 73 °C (starch gelatinisation) and 100.34 °C (protein denaturation), and contains a mixture of protein types and non-protein components such as starch and fibre, resulting in less-efficient protein adsorption and the lowest emulsification capacity. Overall, the results indicate that higher protein purity favours emulsification capacity, whereas greater matrix complexity promotes emulsion stability [62].

#### 4.5.4. Foaming Capacity and Stability of FB Derivatives

Foam consists of a gas dispersed within a continuous liquid phase. Proteins, being amphiphilic and surface-active, stabilise foams by reducing interfacial tension, promoting foam formation, and maintaining bubble stability by creating viscoelastic interfacial films [63,64]. Foam-forming ability is evaluated by foaming capacity (FC), which indicates the increase in volume after whipping. Faba bean protein isolate shows a wide FC range (15–77%), depending on the extraction method [65]. In this study, the FC values (Table 4) were within the range reported in the literature. FBPC exhibited the highest FC among all samples, likely due to better solubility, preservation of native protein structures, less aggregation, and more flexible secondary structures with more unordered or β-turn conformations, as indicated by FTIR results, allowing efficient diffusion to the air–water interface and effective film formation [36,66]. In contrast, FBPI showed lower FC, likely because protein denaturation during alkaline extraction and drying reduced its surface activity and flexibility. Based on comparisons of protein solubility, water-holding capacity, foaming capacity, and stability, FBPI’s low foaming capacity can be directly linked to low protein solubility [36,67,68]. Meanwhile, FBF showed the lowest FC, likely because its high levels of non-protein components interfere with protein–interface interactions and restrict air incorporation [69]. Interestingly, despite its lower foaming capacity, the flour sample exhibited the highest foam stability, whereas the protein isolate showed the lowest. This may be due to the flour’s high starch and soluble fibre content, which increases viscosity and reduces liquid drainage from the foam, thereby enhancing stability [69]. Although the protein isolate is richer in protein, it likely formed brittle interfacial films due to processing-induced denaturation, causing the foam to collapse more quickly [68]. Overall, these results reveal that foaming capacity and stability are governed by different mechanisms and do not necessarily correlate.

#### 4.5.5. Impact of Fractionation on Protein Solubility

Protein solubility (PS) is closely linked to functional properties and serves as a useful indicator of quality in protein powders. It primarily depends on the balance of protein–protein and protein–water interactions, as well as surface charge [5,65]. FBPI had noticeably lower PS than that of either FBPC or FBF. This difference may reflect variations in protein composition, molecular weight, and the solubility of protein subunits (legumin and vicilin), as well as the balance of hydrophobic and hydrophilic properties. More aggregated and ordered secondary structures, such as the increased β-sheet content shown in Table 3, make proteins less flexible and less able to interact with water, thereby reducing solubility [70]. In contrast, FBPC showed intermediate solubility, likely due to a more balanced distribution of secondary structures and less aggregation, which improves water interaction and leads to higher solubility than that of isolates [35,36]. Despite exhibiting the highest aggregation levels and the lowest β-sheet content, FBF achieved the highest protein solubility because of the nature of these aggregates and the disorder in its protein structure. Proteins with less rigid secondary structures, such as α-helices and β-sheets, tend to form more exposed, loosely packed hydrophilic aggregates that allow better water penetration and solubilisation than allowed by the tightly cross-linked aggregates typical of more processed protein isolates [71,72,73]. These findings are consistent with previous reports showing higher solubility for dry-fractionated faba bean protein concentrates (approximately 85%) than for wet-fractionated isolates (approximately 30%) [35,36]. Reduced solubility in isolates has been attributed to alkaline extraction, heating, and drying, which can expose hydrophobic regions and promote irreversible aggregation [67]. Overall, the results indicate that processing-induced changes in protein structure and aggregation play a major role in determining the solubility of faba bean derivatives.

#### 4.5.6. Gelation

Faba bean flour (FBF) formed a relatively firm gel at a significantly lower concentration than that for the faba bean protein concentrates (FBPC) and protein isolates (FBPI) (*p* < 0.05). This disparity likely reflects compositional differences among the samples, particularly the retention of carbohydrates, lipids, and fibre in the whole-flour matrix, which are reduced during protein concentration and extraction. Protein–polysaccharide interactions are known to strengthen gel network formation in legume systems [74]. Consistent with the present findings, LGC values ranging from 10% to 16% have been reported for several plant flours, including lupin, green pea, faba bean, hemp, buckwheat, and wheat, with faba bean flour having one of the lowest LGCs (12%); the authors suggested that higher carbohydrate content contributes to improved gelation behaviour [75]. Similarly, a least gelation concentration (LGC) of 10% has been reported for ultrasound-extracted FBPI at neutral pH [19]. Collectively, these findings suggest that FBF’s superior gelation ability may stem from synergistic interactions between its protein and non-protein components. This is supported by the thermal analysis, where FBF exhibited a denaturation temperature of 73 °C (starch gelatinisation), lower than that of either FBPI or FBPC.

### 4.6. Rheological Properties of Faba Bean Derivatives (Flour, Protein Concentrates and Isolates)

Faba bean rheological properties vary according to protein and starch composition, extraction methods, and processing conditions [19]. Differences in G′ behaviour among FBPI, FBPC, and FBF may stem from compositional differences and the thermal responses of starch and proteins. The rapid increase in G′ values observed in FBF during heating is primarily associated with starch gelatinisation and the formation of a viscoelastic network, while subsequent cooling promotes starch retrogradation and protein reassociation, resulting in further strengthening of the gel structure [76]. FBF exhibited the highest G′ and G″ values, likely because its combination of protein, starch, and fibre acts as an active filler that reinforces the gel network and enhances structural stability [62,77]. In contrast, FBPI showed lower G′ and G″ values during heating than during cooling, likely because partial protein unfolding and aggregation occurred during commercial isolation before thermal analysis [78]. FBPC exhibited intermediate behaviour due to its higher protein content while retaining some starch and fibre, with cooling G′ and G″ values exceeding those of FBF.

The damping factor (tan δ = G″/G′) reflects the balance between viscous and elastic behaviour, with lower values indicating more elastic, solid-like behaviour and higher values indicating greater viscous energy dissipation [56]. Tan δ was the highest for FBPC, followed by FBF, and the lowest for FBPI. The greater starch and fibre content in FBPC and FBF likely increased viscous behaviour during heating, whereas the predominantly protein-based FBPI formed more elastic networks [38,76,79]. However, tan δ values converged during cooling for all samples, suggesting that protein denaturation and gelation dominated final structure formation and produced gels with a comparable elastic-to-viscous balance [76].

Similar compositional effects were observed during frequency sweeps; FBF, which contains substantial amounts of starch and fibre, in addition to protein, forms a complex matrix that enhances elasticity and gel strength at lower frequencies [80]. However, as frequency increased, G′ values for FBF decreased more rapidly than for FBPC and FBPI, indicating structural instability [62]. FBPC and FBPI maintained more stable G′ values, intersecting with FBF at approximately 0. 0.8 Hz and surpassing it at higher frequencies, indicating a shift from starch–fibre network dominance to protein–protein interactions. This behaviour is consistent with reports that polysaccharides in flour contribute to thickening and gelling under dynamic conditions [80,81]. The loss modulus (G″) showed a similar pattern to that of the storage modulus, with FBF exhibiting the lowest values across the frequency range and consequently, the lowest tan δ values, indicating highly elastic, minimally viscous behaviour due to starch gelatinisation. In contrast, FBPC and FBPI, with higher protein and lower starch and fibre contents, formed more flexible networks with greater molecular mobility, resulting in higher G″ and tan δ values. The low tan δ values of FBF are consistent with previous reports for faba bean flour gels (tan δ = 0.11- 0.18), which demonstrate predominantly elastic, solid-like behaviour [76].

These findings demonstrate the distinct structural and functional properties of faba bean ingredients. FBF, largely driven by carbohydrate interactions, forms a rigid but relatively brittle matrix, whereas FBPC and FBPI develop more resilient and adaptable protein-rich networks that may be advantageous in applications requiring elastic stability under mechanical stress, such as plant-based gels, emulsions, and meat analogues. Similar behaviour has been reported in faba bean and other legume gels, where starch and fibre enhance gel elasticity and rigidity at small deformations but may reduce the network’s ability to withstand larger deformations due to structural inhomogeneities [56].

### 4.7. Thermal Properties of Faba Bean Derivatives Differential Scanning Calorimetry

Differences in denaturation temperature (T_d_) and denaturation enthalpy (ΔH) among faba bean ingredients reflected variations in protein structure, purity, and processing history. The presence of two thermal transitions in faba bean flour likely reflects its heterogeneous composition, with the lower-temperature peak corresponding to starch gelatinisation or the denaturation of less-stable protein fractions, such as albumins and certain 7S globulins (vicilin and convicilin) and the higher-temperature peak associated with the denaturation of more-stable proteins, such as legumin and vicilin [39]. The complex flour matrix, composed of proteins, starch, and fibre, causes multiple thermal events, as these components denature at different temperatures. These thermal properties indicate that some proteins in flour denature at lower temperatures than those for isolates or concentrates, affecting gelation and processing [38,46]. Faba bean protein concentrate (FBPC) showed a higher denaturation temperature than that of the protein isolates (FBPI), likely because of residual non-protein components or differences in protein structure and extraction-induced aggregation [82,83]. The elevated T_d_ of FBPC suggests the unfolding of critical storage proteins such as legumin and vicilin [38]. The relatively high enthalpy reflects strong protein interactions and well-structured secondary elements, such as β-sheets and β-turns, which require substantial energy to denature. These characteristics suggest greater thermal stability and potential to withstand high-temperature processing conditions while supporting formation of firm gel networks. FBPI exhibited a narrow T_d_ range, indicating a more uniform protein composition with limited aggregation. Its lower ΔH compared with that of FBPC suggests that less energy was required for denaturation, likely due to partial unfolding or fewer stabilising interactions within the protein structure. Consequently, FBPC appears to retain more native-protein conformations, resulting in greater thermal stability and higher ΔH values. In contrast, more intensive extraction processes can yield less-ordered protein structures with lower ΔH. Although FBF exhibited a relatively high T_d_ (100 °C), associated with legumin, its intermediate ΔH likely resulted from starch and fibre, which can disrupt protein interactions and reduce structural order [46,84]. Previous studies have shown that higher ΔH values are associated with more-ordered and thermally stable protein structures [85], while sharper denaturation peaks indicate a more homogeneous protein matrix [8]. Likewise, higher T_d_ values reflect greater resistance to protein unfolding due to stronger stabilising interactions such as hydrogen bonding, hydrophobic interactions, and covalent cross-links [86]. Overall, our findings are consistent with previous reports showing that protein purity and extraction methods strongly influence the thermal properties of legume proteins, with isolates generally showing lower denaturation temperatures and enthalpies because of reduced structural stabilisation [46,84].

### 4.8. Amino Acid Composition (AA) of Faba Bean Derivatives

The concentrations of all essential amino acids increased significantly (*p* < 0.05) from faba bean flour (FBF) to protein concentrate (FBPC) and protein isolate (FBPI), with the proportion of essential amino acids relative to total amino acids increasing from 40.73% to 41. 63% and 42.68%, respectively. These changes reflect the removal of starch, fibre, and other non-protein constituents during processing, resulting in a protein-rich fraction. The relatively high lysine levels demonstrate the nutritional advantage of faba bean proteins, particularly for complementing cereal-based foods, where lysine is often the limiting amino acid [6]. Moreover, enrichment in branched-chain amino acids (leucine, isoleucine, and valine) enhances the nutritional value of FBPC and FBPI, as these amino acids play important roles in growth, tissue repair, and protein synthesis [87]. Despite the overall increase in essential amino acid concentrations, sulphur-containing amino acids remained the most limiting, indicating that methionine and cysteine remain nutritional constraints in faba bean protein products [7,31]. This limitation was reflected in the amino acid score and the digestible indispensable amino acid score (DIAAS), which, although improved following processing, remained below the ideal reference values. Similar DIAAS values, around 62% for pea protein concentrates and 61% for pea protein isolates, have been reported [30,88]. Given the rising popularity of faba bean use as an ingredient in plant-based foods, a more detailed investigation into the protein quality of faba beans is warranted [89].

## 5. Conclusions

The systematic comparison of faba bean derivatives (flour, protein concentrates, and isolates) in this study highlights their distinct and complementary functional benefits. Faba bean flour outperformed protein concentrates and isolates in regard to solubility, gelation, foaming, and emulsification stability, whereas protein isolates excelled in other functional properties, with protein concentrate generally showing intermediate performance. These findings demonstrate that functional performance does not necessarily improve with protein purification and that non-protein constituents in faba bean flour may contribute to specific techno-functional properties. Importantly, our findings support the functional viability of whole-faba-bean flour, demonstrating that optimising raw materials can reduce reliance on refined ingredients in sustainable food systems. Consequently, the selection of a faba bean derivative should be guided by the functional requirements of the intended food application. The observed differences in hydration, interfacial, thermal, and rheological properties suggest that these derivatives could play complementary roles in food formulations. Faba bean flour may be ideal for applications requiring strong gelation, foaming, and emulsion stability, such as baked foods. In contrast, faba bean protein concentrates and isolates could be better suited for high-protein products that demand specific textures and structural attributes. Collectively, these functional differences highlight the potential suitability of faba bean derivatives for a range of food applications, such as emulsified foods, baked goods, high-protein formulations, and meat analogues, where moisture retention, fat binding, texture, and structural stability are essential qualities. While this study provides valuable insights into the relationship between commercial refinement and ingredient functionality, it is limited to commercially produced ingredients manufactured using proprietary processing methods and cultivars, preventing identification of the precise factors responsible for the observed structural and functional differences. Future studies employing controlled, standardised laboratory-scale processing should investigate how specific fractionation conditions and specific cultivars influence ingredient functionality and performance. By connecting raw ingredient characterisation with advanced texturisation requirements, this study provides a valuable foundation for the targeted selection and optimisation of faba bean ingredients for next-generation sustainable food analogue products.

## Figures and Tables

**Table 1:** Proximate composition (dry basis) of faba bean derivatives.

Composition (%)	FBPI	FBPC	FBF
Crude protein content	87.28 ± 0.25 ^a^	60.47 ± 0.09 ^b^	32.29 ± 0.21 ^c^
Ash content	3.51 ± 0.04 ^b^	6.50 ± 0.05 ^a^	3.35 ± 0.03 ^c^
Fat content	0.67± 0.16 ^c^	3.32 ± 0.06 ^a^	1.50 ± 0.06 ^b^
Total dietary fibre	1.94± 0.06 ^c^	12.95 ± 0.64 ^a^	7.21 ± 0.23 ^b^
Starch	1.13± 0.06 ^c^	5.80 ± 0.10 ^b^	40.13 ± 1.19 ^a^
Total carbohydrates (by difference)	6.59± 0.38 ^c^	16.77 ± 0.61 ^b^	55.65 ± 0.46 ^a^

Note: The data represent the mean ± standard deviation of triplicate analyses. Values with different superscripts within the same row are significantly different (*p* < 0.05). Faba bean flour (FBF); faba bean protein concentrate (FBPC); faba bean protein isolate (FBPI).

**Table 2:** L*, a* and b* colour parameters of raw faba bean derivatives.

Sample		Colour			
	Image	L*	a*	b*	Pairwise ∆E* (Comparison)
FBPI	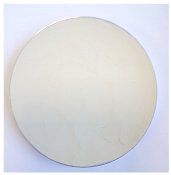	88.71 ± 0.20 ^c^	−0.98 ± 0.03 ^b^	15.16 ± 0.13 ^a^	(FBPI vs. FBPC) 8.38
FBPC	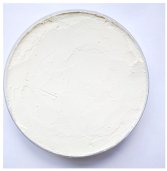	96.77 ± 0.06 ^a^	−1.27 ± 0.02 ^c^	12.89 ± 0.18 ^c^	(FBPC vs. FBF) 4.30
FBF	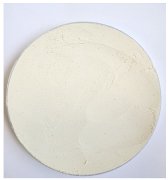	92.64 ± 0.04 ^b^	−0.47 ± 0.05 ^a^	13.76 ± 0.16 ^b^	(FBF vs. FBPI) 4.20

Note: The data represent the mean ± standard deviation of triplicate analyses. Values with different superscripts within the same column are significantly different (*p* < 0.05). Faba bean flour (FBF); faba bean protein concentrate (FBPC); faba bean protein isolate (FBPI).

**Table 3:** Secondary structures of the protein in raw faba bean derivatives.

Sample	α-Helix (%)	β-Sheets (%)	β-Turns (%)	Random Coils (%)	Aggregates (%)
FBPI	12.42 ± 0.08 ^a^	39.11 ± 0.03 ^a^	22.29 ± 0.07 ^a^	20.96 ± 0.15 ^a^	5.22 ± 0.13 ^b^
FBPC	12.47 ± 0.09 ^a^	38.89 ± 0.07 ^b^	22.71 ± 0.24 ^a^	20.89 ± 0.12 ^a^	5.04 ± 0.51 ^b^
FBF	11.66 ± 0.12 ^b^	38.67 ± 0.07 ^c^	22.36 ± 0.25 ^a^	19.60 ± 0.23 ^b^	7.71 ± 0.59 ^a^

Note: The data above represent the mean ± standard deviation of triplicate analyses. Values with different superscripts within the same column are significantly different (*p* < 0.05). Faba bean flour (FBF); faba bean protein concentrate (FBPC); faba bean protein isolate (FBPI).

**Table 4:** Techno-functional properties of faba bean derivatives.

Functional Properties (%)	FBPI	FBPC	FBF
Water-holding capacity (WHC)	601.9 ± 0.1 ^a^	183.3 ± 0.3 ^c^	217.5 ± 0.3 ^b^
Oil-holding capacity (OHC)	380.7 ± 0.4 ^a^	303.3 ± 0.2 ^b^	282.2 ± 0.2 ^c^
Emulsification capacity (EC)	93.2± 0.3 ^a^	58.0 ± 0.3 ^b^	53.8 ± 0.5 ^c^
Emulsification stability (ES)	49.9 ± 0.1 ^c^	71.5 ± 0.8 ^b^	86.7 ± 0.5 ^a^
Foaming capacity (FC)	20.4 ± 0.8 ^b^	23.1± 0.4 ^a^	11.1 ± 0.4 ^c^
Foaming stability (FS)	91.9 ± 0.3 ^c^	94.8 ± 0.3 ^b^	99.0 ± 0.3 ^a^
Least gelation concentration (LGC)	12.0 ± 0.0 ^a^	12.0 ± 0.0 ^a^	8.0 ± 0.0 ^b^
Protein solubility (PS)	43.1 ± 0.5 ^c^	69.0 ± 0.5 ^b^	72.8 ± 0.3 ^a^

Note: The data above represent the mean ± standard deviation of triplicate analyses. Values with different superscripts within the same row are significantly different (*p* < 0.05). Faba bean flour (FBF); faba bean protein concentrate (FBPC); faba bean protein isolate (FBPI).

**Table 5:** Thermal properties of the raw faba bean derivatives.

Sample	Onset Temperature (T_o_)	Denaturation Temperature (T_d_)	End Temperature (T_e_)	Enthalpy Change (ΔH) J/g
FBPI	81.42 ± 0.59 ^b^	89.89 ± 0.28 ^c^	118.13 ± 0.01 ^a^	0.44 ± 0.13 ^c^
FBPC	83.75 ± 1.30 ^b^	97.90 ± 0.13 ^b^	118.10 ± 0.04 ^a^	1.30 ± 0.33 ^a^
FBF	64.32 ± 0.64 ^c^ 93.96 ± 1.07 ^a^	73.06 ± 0.06 ^d^ 100.34 ± 0.06 ^a^	83.54 ± 1.48 ^c^ 111.59 ± 2.67 ^b^	0.86 ± 0.07 ^b^ 0.47 ± 0.21 ^c^

Note: The data above represent the mean ± standard deviation of triplicate analyses. Values with different superscripts within the same column are significantly different (*p* < 0.05). Faba bean flour (FBF); Faba bean protein concentrate (FBPC); Faba bean protein isolate (FBPI).

**Table 6:** Amino acid composition and DIASS results for faba bean derivatives.

AMINO ACIDS (mg/100 mg Sample)	FBF	FBPC	FBPI	Reference [30] (6 Months–3 yrs.)
**Essential Amino Acids (EAA)**				
Histidine	0.82 ± 0.05 ^b^	1.29 ± 0.32 ^b^	2.19 ± 0.09 ^a^	20
Isoleucine	1.28 ± 0.03 ^c^	2.49 ± 0.01 ^b^	3.80 ± 0.06 ^a^	32
Leucine	2.19 ± 0.03 ^c^	4.40 ± 0.06 ^b^	6.62 ± 0.09 ^a^	66
Lysine	2.27 ± 0.09 ^c^	3.96 ± 0.11 ^b^	6.06 ± 0.11 ^a^	57
Methionine	0.27 ± 0.01 ^c^	0.59 ± 0.06 ^b^	1.07 ± 0.08 ^a^	27 (Cys + Met)
Phenylalanine	1.36 ± 0.03 ^c^	2.69 ± 0.03 ^b^	4.01 ± 0.07 ^a^	52 (Phe + Tyr)
Threonine	1.12 ± 0.07 ^c^	2.12 ± 0.05 ^b^	3.20 ± 0.07 ^a^	31
Tyrosine	1.26 ± 0.07 ^c^	2.44 ± 0.10 ^b^	3.65 ± 0.04 ^a^	
Valine	1.55 ± 0.05 ^c^	2.95 ± 0.04 ^b^	4.44 ± 0.06 ^a^	43
**Non-Essential Amino Acids (NEAA)**				
Alanine	1.42 ± 0.08 ^c^	2.46 ± 0.04 ^b^	3.75 ± 0.06 ^a^	
Arginine	3.03 ± 0.14 ^c^	5.96 ± 0.01 ^b^	8.13 ± 0.16 ^a^	
Aspartic acid	3.37 ± 0.13 ^c^	6.23 ± 0.27 ^b^	9.05 ± 0.26 ^a^	
Cysteine	0.30 ± 0.01 ^c^	0.65 ± 0.03 ^b^	0.85 ± 0.04 ^a^	
Glutamic acid	5.10 ± 0.17 ^c^	10.14 ± 0.76 ^b^	15.15 ± 0.60 ^a^	
Glycine	1.53 ± 0.07 ^c^	2.54 ± 0.17 ^b^	3.43 ± 0.08 ^a^	
Proline	1.52 ± 0.08 ^c^	2.74 ± 0.03 ^b^	3.99 ± 0.05 ^a^	
Serine	2.09 ± 0.39 ^c^	2.97 ± 0.09 ^b^	4.66 ± 0.12 ^a^	
Taurine	0.04 ± 0.00 ^a^	0.02 ± 0.01 ^b^	0.03 ± 0.00 ^b^	
**% EAA/TAA**	40.73	41.63	42.68	
**AAS**	66.14	76.16	81.47	
**DIAAR (100% Digest)**	0.61	0.55	-	
**DIAAS (Adjusted % Digestibility)**	61.12 SAA	55.38 SAA	-	

Note: The data above represent the mean ± standard deviation of triplicate analyses. Values with different superscripts within the same row are significantly different (*p* < 0.05). Faba bean flour (FBF); faba bean protein concentrate (FBPC); faba bean protein isolate (FBPI). Abbreviations: % EAAs/TAA, essential amino acids/total amino acids; AAS, amino acid score; DIAAR, digestible indispensable amino acid ratio; DIAAS, digestible indispensable amino acid score; SAA, sulphur amino acids. Faba bean flour (FBF); faba bean protein concentrate (FBPC); faba bean protein isolate (FBPI). DIAA ratios and DIAAS were calculated using the recommended scoring pattern [30].

## Data Availability

The original contributions presented in this study are included in the article/Supplementary Material. Further inquiries can be directed to the corresponding author.

## Supplementary Materials

The following supporting information can be downloaded at: https://www.mdpi.com/article/10.3390/foods15213987/s1, Figure S1: Strain sweeps for commercial faba bean derivatives; (A) protein isolates (FBPI), (B) protein concentrates (FBPC), (C) flour (FBF).
