# Supplementary material for: Impact of Refinement on the Physicochemical, Structural, Functional, and Nutritional Properties of the Faba Bean (Vicia faba L.)
Source: Foods. 2026 Nov 6;15(21):3987. doi: 10.3390/foods15213987 (PMC13762758; doi:10.3390/foods15213987)
Supplement: Supplementary file 1 [file foods-15-03987-s001.zip › foods-4573742-supplementary.pdf]

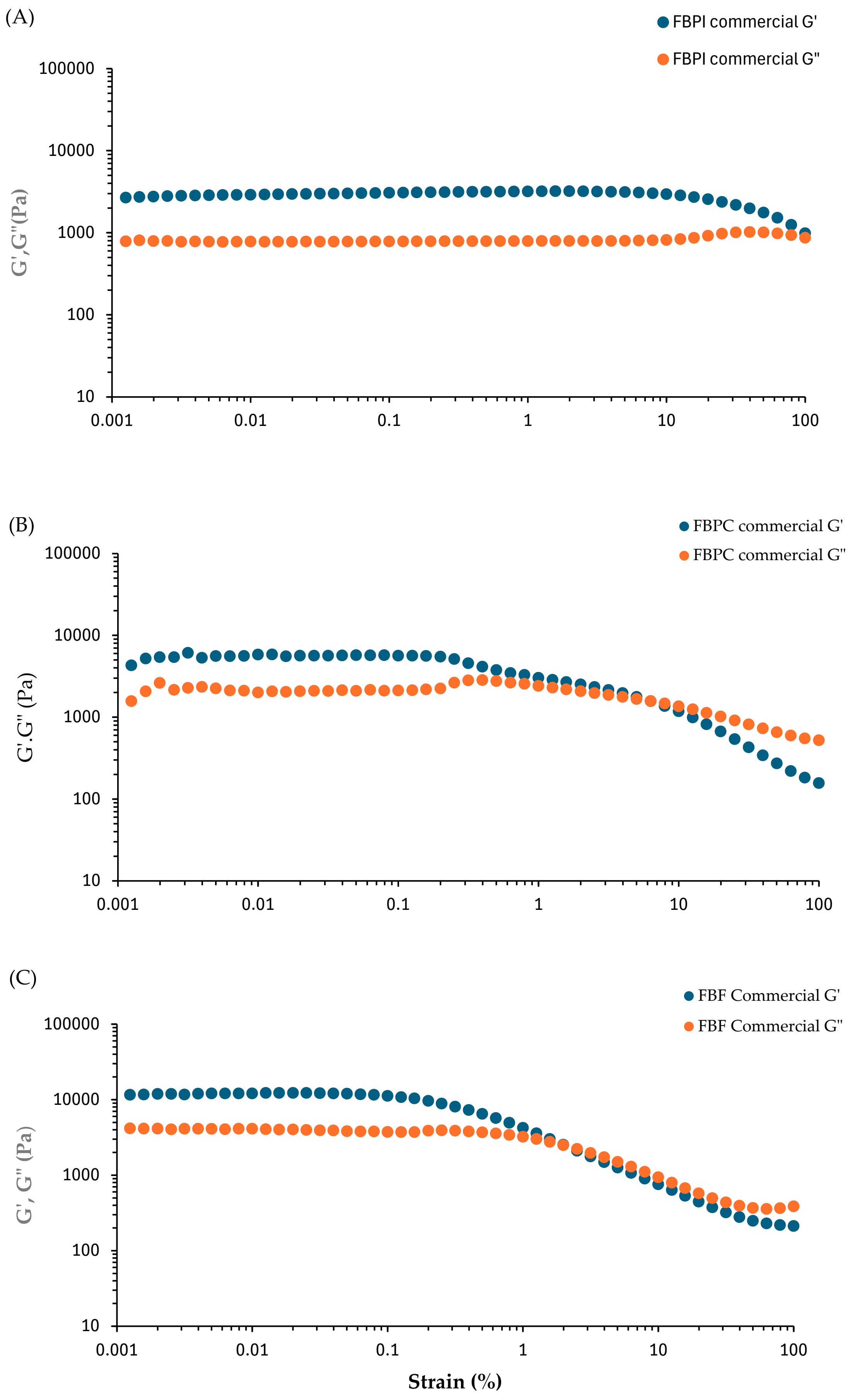


**Figure S1**: Strain sweeps for commercial faba bean derivatives; (A)protein isolates (FBPI), (B) protein concentrates (FBPC), (C) flour (FBF).
